# Response to comment on human cytomegalovirus seropositivity is associated with reduced patient survival during sepsis

**DOI:** 10.1186/s13054-023-04756-4

**Published:** 2023-11-28

**Authors:** Björn Koos, Matthias Unterberg, Tim Rahmel, Michael Adamzik, Stefan F. Ehrentraut

**Affiliations:** 1 Klinik für Anästhesiologie, Intensivmedizin und Schmerztherapie, Universitätsklinikum Knappschaftskrankenhaus Bochum, Bochum, Germany; 2 Klinik für Anästhesiologie und Operative Intensivmedizin, Universitätsklinikum Bonn, Bonn, Germany

Dear Editor:

We read the comment by Drs. Wang and Zhong to our article *Human cytomegalovirus seropositivity is associated with reduced patient survival during sepsis* with great interest [1]. While we agree with the authors that more work is needed to further study the exciting findings of our article, they suffer from some serious misconceptions.

First and foremost, the authors seem to conflict HCMV seropositivity with HCMV re-activation, as their entire comment focusses on re-activation. The impact of HCMV re-activation (also called HCMV DNAemia) on sepsis outcome is a hotly discussed topic in the field with interesting studies supporting different views [2, 3]. Our work in contrast does not focus on re-activation, but rather on seropositivity of HCMV. While re-activation is defined as a re-emergence of viral DNA in the blood of the patient (hence, DNAemia [4]), HCMV seropositivity is generally defined as the patient being positive for HCMV IgG antibodies resulting from a previous HCMV infection [5, 6]. HCMV DNA cannot be detected in the blood of these patients, as long as they show no signs of re-activation.

HCMV, as all herpes viruses, stays in the body of the host after the primary infection in a state of latency. Our work now shows that this latency, independently of re-activation, has a detrimental effect on survival. While we cannot exclude the possibility of later re-activation, the effect of latency seems to be responsible for our findings [1].

Following their line of thought, Drs. Wang and Zhong point out that we show a much lower rate of HCMV re-activation compared to other studies [4, 7]. Unfortunately, this as well is not completely accurate. While generally studies show a re-activation rate of 20–70% in sepsis and COVID-19 [4], this re-activation occurs later in the disease progression. For example, the study of Gatto et al*.*, Drs. Wang and Zhong refer to, reports a median re-activation time of 17 days [4]. As we only checked for re-activation until day 8 after sepsis onset, we will miss any re-activation that might occur later. This is a limitation of our study, but more importantly, we did not focus on re-activation but on latency (or seropositivity) of HCMV at day 1 of sepsis onset. Furthermore, our main finding, latency’s impact on survival becomes obvious and significant before day 8 making a confounding effect of re-activation after day 8 less important. Moreover, our findings demonstrate comparable re-activation levels of HCMV at 7.5% [1], in comparison with the 4% reported by Gatto et al. upon ICU admission [4].

To their second point: The authors point out that early death skews the incidence of HCMV re-activation. The rationale being that patients that die in early stages of the disease do not have time to re-activate. This is an interesting approach, and we did not correct for this. However, we would like to point out again that we did not focus on HCMV re-activation but on HCMV latency (or seropositivity).

In their last point, the authors suggest to study the re-activation of other herpesviruses in sepsis, since the authors themselves have shown the re-activation of multiple herpesviruses to be of importance [8]. We agree with this assessment. However, we do not see the relevance to our article as we focused our work on HCMV latency (or seropositivity) and not on re-activation.

## Data Availability

No datasets were generated or analysed during the current study.
